# Tartary Buckwheat Flavonoids Improve Colon Lesions and Modulate Gut Microbiota Composition in Diabetic Mice

**DOI:** 10.1155/2022/4524444

**Published:** 2022-08-16

**Authors:** Wenwen Cheng, Cifeng Cai, Ivan Kreft, Tamara Lah Turnšek, Mingyan Zu, Yongping Hu, Meiliang Zhou, Zhiyong Liao

**Affiliations:** ^1^College of Life and Environmental Science, Wenzhou University, Wenzhou 325035, China; ^2^University of Ljubljana, Jamnikarjeva 101, Ljubljana SI-1000, Slovenia; ^3^National Institute of Biology, Večna Pot 111, Ljubljana SI-1000, Slovenia; ^4^Yantai Jinrui Female Products Co., Ltd, Yantai 264000, China; ^5^Weining Dongfang Shengu Co., Ltd, Guizhou 553100, China; ^6^Institute of Crop Science, Chinese Academy of Agricultural Sciences, Beijing100081, China

## Abstract

Tartary buckwheat flavonoids (TBFs) exhibit diverse biological activities, with antioxidant, antidiabetes, anti-inflammatory, and cholesterol-lowering properties. In this study, we investigated the role of TBFs in attenuating glucose and lipid disturbances in diabetic mice and hence preventing the occurrence of diabetes-related colon lesions in mice by regulating the gut microbiota. The results showed that TBFs (1) reversed blood glucose levels and body weight changes; (2) improved levels of serum total cholesterol (TC), triglycerides (TGs), low-density lipoprotein cholesterol (LDL-C), high-density lipoprotein cholesterol (HDL-C), tumor necrosis factor-*α* (TNF-*α*), interleukin-6 (IL-6), and fasting insulin; and (3) significantly reduced diabetes-related colon lesions in diabetic mice. In addition, TBFs also affected the diabetes-related imbalance of the gut microbiota and enriched beneficial microbiota, including *Akkermansia* and *Prevotella.* The TBF also selectively increased short-chain fatty acid-producing bacteria, including *Roseburia* and *Odoribacter*, and decreased the abundance of the diabetes-related gut microbiota, including *Escherichia*, *Mucispirillum*, and *Bilophila*. The correlation analysis indicated that TBFs improved metabolic parameters related to key communities of the gut microbiota. Our data suggested that TBFs alleviated glucose and lipid disturbances and improved colon lesions in diabetic mice, possibly by regulating the community composition of the gut microbiota. This regulation of the gut microbiota composition may explain the observed effects of TBFs to alleviate diabetes-related symptoms.

## 1. Introduction

In recent years, the incidence of diabetes has dramatically increased worldwide. Diabetes is a metabolic disorder disease with multiple potential causes and is characterized by chronic high blood glucose, due to insulin deficiency or insulin resistance [1]. Although some antidiabetes drugs have been tested for clinical use, there are few effective approaches to prevent the initiation and development of diabetes and obesity [2]. Colon lesions appear in the initial phase of diabetes-induced colon cancer, and at present, there are no treatments available to prevent the development of colon lesions, due to the poor knowledge of this process. Several studies have indicated that the pathogenesis of diabetes is closely related to the gut microbiota, which can affect host metabolism, immune regulation, the development of inflammatory bowel disease, and chronic inflammation [3–7]. The composition of the gut microbiota is also correlated with obesity [8–13]. With escalating incidence of diabetes, synthetic insulin and antidiabetic agents are clinically administered for diabetic therapy, but these drugs present side effects, toxicity, and high costs [14]. Therefore, it is essential to develop novel and effective drug leads for the treatment of diabetes. There is a growing interest in the development of natural products for diabetes treatment, as these compounds may have minimal side effects.

Tartary buckwheat is a species of the Polygonum family, with a high nutritional value and a nutritionally balanced composition of proteins, vitamins, and flavonoids [15]. Tartary buckwheat flavonoids (TBFs) contain mostly rutin and quercetin. TBFs exhibit antioxidant, free radical scavenging, anti-aging, antidiabetic, anti-inflammatory, and cholesterol-lowering activities. These biological properties have been attributed to the presence of multiple flavonoids, including rutin, quercetin, and kaempferol [16–21]. Recent studies have indicated that the regulation of gut microbiota may partially explain the antidiabetic effects of natural products [22]. However, it still remains unknown whether TBFs can improve diabetes and colon lesions by modulating the gut microbiota.

In this study, we investigated the effect of TBFs to mitigate the diabetes pathological state and diabetes-related colon lesions. Furthermore, we investigated the ability of TBF treatment to regulate gut microbiota dysbiosis in diabetic mice. The effects of TBF on colon tissue damage and gut microbiota composition were analyzed in diabetic mice to explore the potential mechanism for the TBF treatment of diabetes. The ability of TBFs to reduce colon lesions and modulate the gut microbiota composition in diabetic mice provides support for the therapeutic potential of TBFs to treat diabetes-related symptoms by the promotion of the intestinal health.

## 2. Materials and Methods

### 2.1. Materials

Streptozotocin, saline, rutin, and quercetin were purchased from Sigma-Aldrich Co., Ltd. (St. Louis, MO, USA). Tartary buckwheat flour was obtained from Weining Dongfang Shengu Co., Ltd. (Guizhou, China). The AB-8 macroporous resin was purchased from Soledad Technology Ltd. (Beijing, China). All other chemical reagents used in this study were of analytical grade or higher.

### 2.2. Extraction of TBF

Briefly, 20 g of Tartary buckwheat flour was extracted with 120 ml of ethanol water (65%, v/v) at 75°C with stirring for 5 h. The extract was filtered using a circulating water multipurpose vacuum pump and then concentrated using a rotary evaporator. The extract was dissolved in 30% ethanol and then purified using AB-8 macroporous adsorption resin. The extraction solution was concentrated under reduced pressure and then dried under vacuum at 50°C. The resulting fraction of purified Tartary buckwheat flavonoids was designated as TBF.

### 2.3. Determination of the TBF Components

The components of the TBF fraction were determined as described elsewhere [23]. The TBFs were analyzed using a high-performance liquid chromatography (HPLC) system (Agilent 1260 LC, Agilent Technology Co., Ltd.) equipped with an Agilent TC-C18 column (4.6 mm × 250 mm, Agilent Technology Co., Ltd.). The sample was eluted using mobile phase A of water and mobile phase B of a water solution containing 95% (v/v) methanol. A linear gradient program was applied as follows: 20% (v/v) B for 0–5 min, 20–40% (v/v) B for 5–15 min, and 40% (v/v) B for 15–40 min. The detection wavelength was set at 260 nm. Pure rutin and quercetin were used as standards for HPLC analysis.

### 2.4. Experimental Animals

Forty healthy male C57BL/6 mice (8 weeks old) were obtained from Shanghai SLAC Laboratory Animal Co., Ltd. The mice were provided with *ad libitum* access to food and water for a week. Mice were kept at SPF-level barriers in the Animal Laboratory Center in Wenzhou Medical University. All mice were raised in separate cages under conditions in which they could freely ingest available solid feed and water. All animal experiments were approved by the Ethical Committee of Animal Health and Care of Wenzhou Medical University, and all experiments were carried out in accordance with the guidelines of laboratory animal welfare ethics and daily animal care guidelines.

### 2.5. Diabetic Mice Model and Experimental Design

After one-week acclimatization, the STZ (streptozotocin)-induced T1DM (type 1 diabetes mellitus) and HFD (high-fat diet)/STZ-induced T2DM (type 2 diabetes mellitus) mice models were established as previously described with some modifications [24]. Briefly, the mice received intraperitoneal injections of STZ (45 mg/kg/day) for five consecutive days. Mice used as the control group were injected with an equivalent volume of citrate buffer (pH 4.4, concentration 0.1 mol/L). Four weeks after diabetes induction, fasting blood glucose level of 16.7 mM and above were considered diabetic. To create the HFD/STZ-induced T2DM mice model, the ND (nondiabetic) group mice were fed a normal diet (10% lipids, 19% protein, 71% carbohydrates) and the other three groups of mice were fed the HFD (45% lipids, 19% proteins and 36% carbohydrates, research diets incorporated company, New Brunswick, New Jersey, USA) for eight weeks. After eight weeks of feeding, the mice were fasted for 12 h overnight. The ND group was treated with a buffer solution, and the other groups were received three days (once a day) intraperitoneal injections of STZ (35 mg·kg−1, dissolved at 0.1 mol/L cold citrate buffer, pH 4.4) to induce T2DM. The fasting blood glucose levels were measured seventy-two hours after the final injection [25]. Fasting mice blood glucose value ≥ 16.7 mmol/L was defined as diabetes for two consecutive days. The dosage of TBF was optimized based on previous experiments [26]. Mice were administered intragastrically with TBF or saline once a day for four weeks as follows: ND group (ND mice, daily administration of saline, *n* = 6), T1DM group (T1DM mice, daily administration of saline, *n* = 5), T1DM-TBF150 group (T1DM mice, daily administration of 150 mg TBF/kg body weight, *n* = 5), T1DM-TBF300 group (T1DM mice, daily administration of 300 mg TBF/kg body weight, *n* = 5), T2DM group (T2DM mice, daily administration of normal saline, *n* = 5), T2DM-TBF150 group (T2DM mice, daily administration of 150 mg TBF/kg body weight, *n* = 5), and T2DM-TBF300 group (T2DM mice, daily administration of 300 mg TBF/kg body weight, *n* = 5). The body weight and fasting blood glucose concentrations were recorded weekly for four weeks, and then, fresh fecal samples were collected under a sterile environment and snap-frozen at −80°C for subsequent analysis. Venous blood samples from each mouse were obtained by cardiac puncture. Supernatants were separated by centrifuging at 3000 rcf (×g) for 15 minutes at 4°C. Samples of colon tissues and intestinal contents were collected, frozen in liquid nitrogen, and stored at −80°C.

### 2.6. Biochemical Analysis

Total cholesterol (TC), triglyceride (TG), low-density lipoprotein cholesterol (LDL-C), and high-density lipoprotein cholesterol (HDL-C) in serum were measured using specific detection kits (Nanjing Jiancheng Bio-Engineering Institute Co., Ltd.). Levels of TNF-*α*, IL-6, and insulin in serum were measured using specific detection kits (Hangzhou MultiSciences Biotech Co., Ltd.). The homeostatic model assessment of insulin resistance (HOMA-IR) was calculated as (fasting blood glucose [mmol/mL] × insulin assay [microunit/mL])/22.5, and the homeostatic model assessment of beta-cell function (HOMA-B) was calculated as (fasting insulin [mU/L] × 20)/(fasting glucose [mmol/L] − 3.5) [27].

### 2.7. Haematoxylin and Eosin (H&E) Staining

The colon tissues were fixed in paraformaldehyde solution (4%) for 48 h at room temperature after washing with saline. All samples were then dehydrated with ethanol and embedded in paraffin. The tissues were cut into 5-*μ*m-thick microsections (3–5 sections/specimen) and subjected to H&E staining. Samples were sealed with neutral resin and observed under an optical microscope (×200 magnification) in 10–20 randomly selected fields of view. The histopathological scores of colonic lesions were based on the following parameters: the degree of inflammation, crypt damage, the number of vacuoles, and villi length. The final pathological scores were calculated by adding all the scores of each parameter (0, no damage; 1, less than 25% damage; 2, 25–50% damage; 3, 50–75% damage; 4, more than 75% damage).

### 2.8. Gut Microbiota Analysis

After four weeks of treatment, 200 mg of fresh fecal samples per mice were collected using sterile swabs and transferred to sterile Eppendorf tubes in a sterile environment. A rapid DNA spin extraction kit (MP biomedicals, Santa Ana, CA, USA) was adopted to extract total bacterial genomic DNA from the fecal samples according to the manufacturer's instructions. DNA was detected by agarose gel electrophoresis and Nanodrop (Thermo Scientific, NC2000). The DNA was amplified with specific bacterial primers targeting the 16S rRNA gene containing the V3-V4 region using universal primers 338F (5′-ACTCCTACGGGAGGCAGCA-3′) and 806R (5′-GGACTACHVGGGTWTCTAAT-3′). PCR products were purified using Agencourt AMPure Beads (Beckman Coulter, Indianapolis, IN) and quantified using the PicoGreen dsDNA assay kit (Invitrogen, Carlsbad, CS, USA). Sequencing was performed by Shanghai Personal Biotechnology Co., Ltd. (Shanghai, China) using the Illumina MiSeq platform to generate 2 × 250 bp paired-end reads. The unique reads were clustered according to the distance between the sequences. Uparse software (v7.0.1001) was used for sequence analysis, and sequences were classified into operational taxonomic units (OTUs) with 97% similarity cutoff after quality filtering and merging. QIIME was applied to calculate the *α *-diversity indices, including the Chao1, Shannon, and Simpson indices. UniFrac distance measurement and principal coordinate analysis (PCoA) were selected for *β*-diversity analysis of the gut microbiota structure, and heatmap analysis was performed using R statistical software (R version 3.1.0: Foundation for Statistical Computing, Vienna, Austria).

### 2.9. Statistical Analysis

Data analysis was completed with GraphPad Prism version 6.00 for Windows (GraphPad Prism Software, San Diego, California, USA, https://www.graphpad.com). All values were expressed as the means ± standard error of measurement (SEM). One-way ANOVA was used for statistical comparisons of different groups. Spearman's correlation analysis was performed using R statistical software (R version 3.4.0: Foundation for Statistical Computing, Vienna, Austria) [28], and a clustering heatmap of correlation coefficients was calculated by Ward' s hierarchical. A value of *p* < 0.05 was considered statistically significant.

## 3. Results

### 3.1. TBF Alleviated Glucose and Lipid Disturbances in Diabetic Mice

We extracted TBF and analyzed its components using HPLC. The prepared TBF appeared to contain 80.67% rutin and 3.33% quercetin (detailed data are shown in Supplementary Figure S1). We next tested the effect of TBF on blood glucose and lipids in STZ (streptozotocin)-induced T1DM (type 1 diabetes mellitus) and HFD (high-fat diet)/STZ-induced T2DM (type 2 diabetes mellitus) mice models. A previous study reported that hyperlipidemia and dyslipidemia are common lipid metabolic syndromes in diabetic patients [29], and both hyperlipidemia and dyslipidemia were observed in diabetic mice. Our results showed that diabetic mice models were successfully established, displaying significantly elevated fasting blood glucose and decreased body weight in diabetic mice, and these changes were significantly reversed by TBF treatment. The concentrations of TC, TG, and LDL-C were significantly increased in diabetic mice, and HDL-C levels were decreased when compared to the ND group, and TBF treatment could restore these changes. The fasting insulin levels were significantly decreased in the T1DM group compared with the level in the ND group, and the levels were increased in T1DM-TBF150 and T1DM-TBF300 groups compared with the level in the T1DM group mice. The fasting insulin levels were significantly increased in the T2DM group compared with the levels in the ND group, and the levels in T2DM-TBF150 and T2DM-TBF300 groups were decreased compared with the levels in the T2DM group mice. Insulin resistance (HOMA-IR) was significantly increased in the T2DM group compared with the ND group and was significantly decreased in T2DM-TBF150 and T2DM-TBF300 groups compared to the level in the T2DM group. Beta-cell function (HOMA-B) was significantly decreased in the T2DM group compared with the ND group, and the concentrations of serum TNF-*α* and IL-6 in both the T1DM group and the T2DM group were significantly increased compared with the levels in the ND group, and these changes were significantly reversed by TBF treatment, as shown in Tables 1–4. Our results revealed that TBF could effectively improve blood glucose metabolism and lipid metabolism in diabetic mice.

### 3.2. TBF Improved Colon Lesions in Diabetic Mice

**Figure 1:**
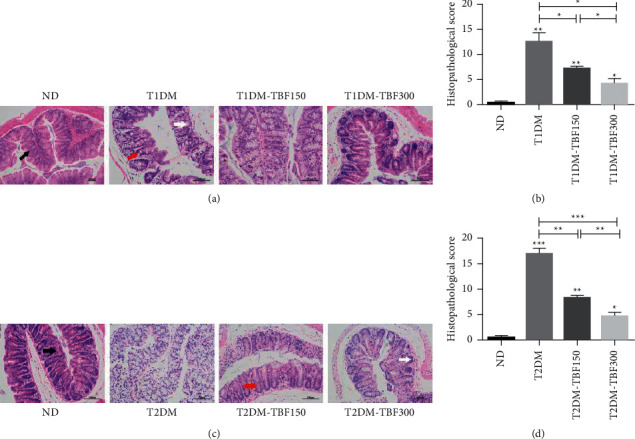
The TBF improved colon lesions in diabetic mice. (a) H&E staining of colon tissues of mice in ND, T1DM, T1DM-TBF150, and T1DM-TBF300 group (scale bar: 100 *μ*m). Red arrows: vacuole; black arrows: villi; white arrows: crypt. (b) Histopathological scores of colon tissues in T1DM mice using H&E staining. (c) H&E staining of colon tissues of mice in ND, T2DM, T2DM-TBF150, and T2DM-TBF300 groups (scale bar: 100 *μ*m). Red arrows: vacuole; black arrows: villi; white arrows: crypt. (d) Histopathological scores of colon tissues in T2DM mice by H&E staining. One-way ANOVA was used for statistical comparisons of diﬀerent groups. Values are mean ± SEM: ^*∗*^*p* < 0.05, ^∗∗^*p* < 0.01, ^∗∗∗^*p* < 0.001.

Obese and diabetic mice with colorectal lesions are at greater risk for the occurrence and development of colorectal cancer [30]. To investigate the potential effect of TBF on colonic lesions in diabetic mice, we used H&E staining. Compared with the ND group, the villi of colon tissues in the T1DM group exhibited a disordered arrangement, with many vacuolar changes and damaged crypts in T1DM groups. TBF administration rescued the damaged villi in colon tissues. Compared with the T1DM group, there were fewer necrotic sites and vacuolar changes and lower colon injury scores in the T1DM-TBF300 group, as shown in Figures 1(a) and 1(b). Compared with the ND group, the T2DM group exhibited abnormal colon tissue morphology, with large amounts of vacuolar changes and damaged crypts in the T2DM groups. TBF administration rescued the damaged villi and the abnormal colon tissue morphology in colon tissues. Compared with the T2DM group, there were fewer necrotic sites and vacuolar changes, as well as lower colon injury scores in the T2DM-TBF300 group. The reduced colon villi/lesions damage observed in both the T1DM-TBF300 group and the T2DM-TBF300 group was more pronounced in the T1DM-TBF150 and T2DM-TBF150 groups, as shown in Figures 1(c) and 1(d). These results indicated that TBF could repair colon lesions caused by diabetes in a dose-dependent manner.

### 3.3. TBF Changed the Overall Community Structure of Gut Microbiota in Diabetic Mice

**Figure 2:**
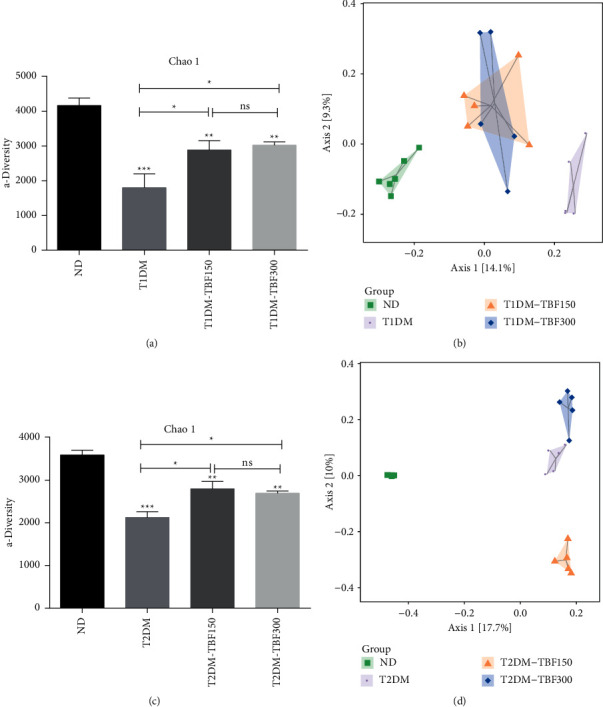
The TBF altered the gut microbiota diversity in diabetic mice. (a) The Chao 1 index in the *α*-diversity analysis in T1DM mice. (b) The *β*-diversity analysis in the ND, T1DM, T1DM-TBF150, and T1DM-TBF300 groups. (c) The Chao 1 index in *α*-diversity analysis in T2DM mice. (d) The *β*-diversity analysis in the ND, T2DM, T2DM-TBF150, T2DM-TBF300 groups. One-way ANOVA was used for statistical comparisons of different groups. Values are means ± SEM: ^*∗*^*p* < 0.05, ^∗∗^*p* < 0.01, ^∗∗∗^*p* < 0.001.

To evaluate the potential effect of TBF on the gut microbial community, fecal samples were collected from the experimental mice. The V3-V4 region of bacterial 16S rRNA was sequenced to determine the overall community structure of gut microbiota in diabetic mice in the presence and absence of TBF treatment. For the experiment with T1DM model mice, 2,840,758 original sequences were obtained in total, with an average of 135,274 ± 13,492 sequences acquired for each sample. After quality screening, a total of 2,347,312 sequences remained, with an average of 111,776 ± 14,983 sequences for each fecal sample. The abundance grade curves and species accumulation curves were constructed, and sufficient gut microbial diversity was captured in each sample at this sequencing depth (detailed data are shown in Supplementary Figure S2). The *α*-diversity analysis revealed that the Chao1 index was significantly lower in the T1DM group than that in the ND group; however, the Chao1 diversity index was significantly increased after TBF administration, as shown in Figure 2(a). The Simpson index was not significantly different among the groups, while the Shannon diversity index was significantly decreased in the T1DM group compared with the ND group, but these differences disappeared with TBF treatment (detailed data are shown in Supplementary Figure S4). The *β*-diversity analysis revealed differences in the gut microbiota in the ND, T1DM, T1DM-TBF150, and T1DM-TBF300 groups, suggesting different microbiota structures among those groups, but the gut microbiota community after TBF treatment did not recover to the ND levels, as shown in Figure 2(b). For the experiment with T2DM model mice, we obtained a total of 2,088,692 original sequences, with an average of 104,434 ± 9,314 sequences per sample. After quality screening, 1,621,546 effective sequences remained, with an average of 81077 ± 8214 sequences for each sample. The abundance grade curves and species accumulation curves were constructed (detailed data are shown in Supplementary Figure S3). The *α*-diversity analysis showed that the Chao1 index was significantly decreased in the T2DM group, indicating lower gut microbiota diversity, and this trend was reversed by the TBF treatment, as shown in Figure 2(c). There was no significant difference in the Simpson index among different groups. The Shannon index was significantly decreased in the T2DM group, whereas dramatically increased after TBF administration (detailed data are shown in Supplementary Figure S4). The *β*-diversity analysis showed that the gut microbiota was clearly distinguished in ND, T2DM, T2DM-TBF150, and T2DM-TBF300 groups, suggesting different microbiota among those four groups, but the gut microbiota community after TBF treatment was different from that in the ND mice, as shown in Figure 2(d). The results showed that TBF changed the overall community structure of gut microbiota in both T1DM and T2DM mice.

### 3.4. TBF Regulated the Composition of Gut Microbiota in Diabetic Mice

**Figure 3:**
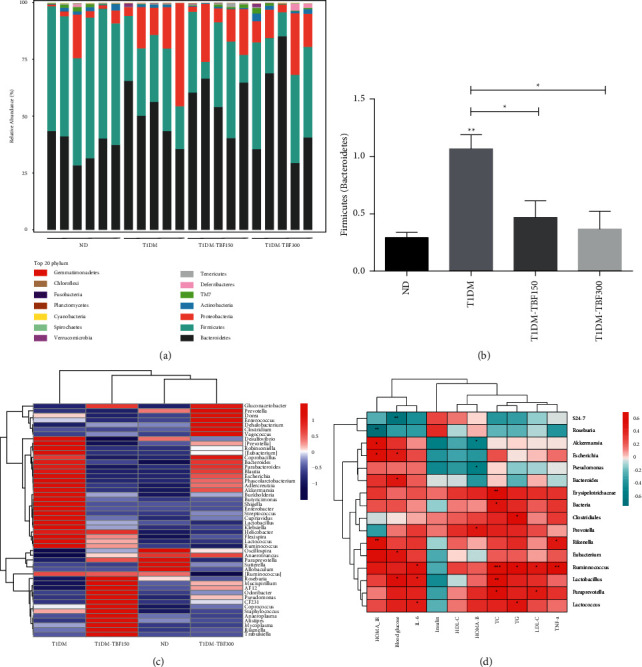
The TBF reconstructed the gut microbiota composition in T1DM mice. (a) The relative abundances of gut microbiota at the phylum level. (b) *Firmicutes*/*Bacteroidetes* in feces of mice at the phylum level. (c) The heatmap of the relative abundances of the gut microbiota at the genus level. Each column in the plot represents a sample, and each row represents the community structure. The colors represent the relative abundances of the species. (d) The heatmap of Spearman's correlation analysis between relative abundances (RAs) of bacterial genera and T1DM mice phenotypes. The colors represent Spearman's correlation co-efficiency: red colors indicate positive relationships and green colors indicate negative relationships. One-way ANOVA was used for statistical comparisons of diﬀerent groups. Values are means ± SEM: ^*∗*^*p* < 0.05, ^∗∗^*p* < 0.01, ^∗∗∗^*p* < 0.001.

We next investigated the composition of gut microbiota in the different treatment groups. Consistent with the findings of a previous report [31], our results indicated that gut microbiota at the phylum level in T1DM model mice were mainly dominated by *Firmicutes* and *Bacteroides*, which together accounted for about 85.2% of the gut microbiota, as shown in Figure 3(a). Our data showed that the *Firmicutes*/*Bacteroidetes* (F/B) ratio in the T1DM group was significantly higher than that in the ND group, and F/B ratios in the T1DM-TBF150 and T1DM-TBF300 groups were lower than those in the T1DM group. Interestingly, the abundances of *Firmicutes* and *Bacteroidetes* in the T1DM-TBF300 group were comparable to the levels in the ND group, as shown in Figure 3(b). At the order level, the relative abundances of *Lactobacillales*, *Campylobacterales*, and *Coriobactales* were increased in the T1DM group compared with the ND group, and these differences disappeared with TBF administration. Furthermore, TBF increased the relative abundances of *Bacteroidales* and *Rickettsiales* that were decreased in diabetic mice (detailed data are shown in Supplementary Figure S5). At the family level, compared with the ND group, the relative abundances of *Erysipelototrichaeae*, *Coriobacteriaeae*, *Streptocaceae*, *Lactobacillaceae,* and *Helicobacteraceae* were increased in the T1DM group, and these changes were reversed by TBFs. Additionally, TBFs increased the relative abundances of *S24-7*, *Lachnospiraceae* and *Prevotellaceae*, which were decreased in diabetic mice (detailed data are shown in Supplementary Figure S6). At the genus level, the relative abundance of gut microbiota in each group was analyzed by heatmap clustering, as shown in Figure 3(c). Compared with the ND group, the relative abundances of *Dorea*, *Helicobacter*, *Escherichia*, *Lactococcus*, and *Streptococcus* were increased in the T1DM group, and these trends were reversed by TBF, with TBF treatment restoring abundances to the levels of the ND group. TBF also increased the relative abundances of *Prevotella*, *Dehalobacterium*, *Clostridium*, *Paraprevotella*, *Roseburia*, *Odoribacter*, and *Rikenella*, bacteria that are negatively correlated with diabetes. Of note, we found that the gut microbiota clustered together according to their correlations with the main metabolic parameters, as shown in Figure 3(d). Blood glucose and HOMA-IR were positively correlated with the relative abundance of *Escherichia*, the relative abundance of *Akkermansia* was negatively correlated with HOMA-B, the relative abundances of *Ruminococcus*, *Lactobacillus,* and *Lactococcus* were positively correlated with TC, TG, LDL-C, TNF-*α*, and IL-6 levels, and abundances of *Lactobacillus* and *Lactococcus* were negatively associated with insulin.

**Figure 4:**
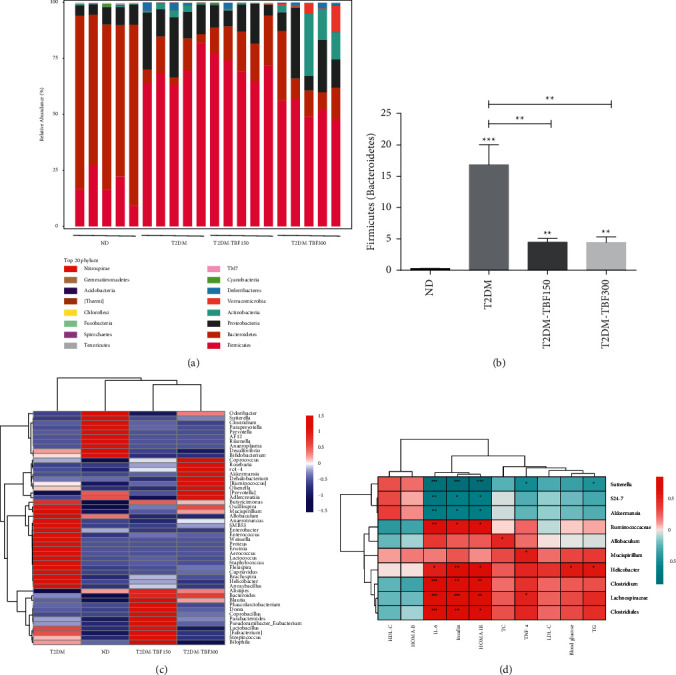
The reconstruction of the gut microbiota composition in T2DM mice after TBF treatment. (a) The relative abundances of gut microbiota at the phylum level. (b) *Firmicutes/Bacteroidetes* in feces of mice at the phylum level. (c) The heatmap of the relative abundances of gut microbiota at the genus level. (d) Each column in the plot represents a sample, and each row represents the community structure. The colors represent the relative abundances of the species. (e) The heatmap of Spearman's correlation analysis between relative abundances (RAs) of bacterial genera and T2DM mice phenotypes. The colors represent Spearman's correlation co-efficiency: red colors indicate positive relationships, and green colors indicate negative relationships. One-way ANOVA was used for statistical comparisons of diﬀerent groups. Values are means ± SEM: ^*∗*^*p* < 0.05, ^∗∗^*p* < 0.01, ^∗∗∗^*p* < 0.001.

For T2DM model mice, at the phylum level, *Firmicutes* and *Bacteroidetes* accounted for about 78.4% of microbiota in the T2DM group, as shown in Figure 4(a). The F/B ratio in the T2DM group was higher than that in the ND group. However, TBF significantly reversed this change, but the abundances of *Firmicutes* and *Bacteroidetes* after TBF treatment were not restored to the levels of the ND group, as shown in Figure 4(b). At the order level, the relative abundances of *Erysipelototrichales*, *Latobacillales*, *Campylobacterales*, and *Staphylococaceae* were increased in the T2DM group, while these bacteria abundances were decreased after TBF. The relative abundances of *Bacteroidales* and *Coribacteriales* were increased by TBF (detailed data are shown in Supplementary Figure S7). At the family level, compared with the ND group, the relative abundances of *Erysipelototrichaeae*, *Streptocaceae*, and *Helicobacteraceae* were increased in the T2DM group, and these changes were reversed by TBFs. However, TBF exhibited limited effect on some decreased gut microbiota in diabetic mice, such as *S24-7*, *Verrucomicrobiaceae* and *Bacteroidaceae* (detailed data are shown in Supplementary Figure S8). At the genus level, compared with the ND group, the relative abundances of *Helicobacter*, *Mucispirillum*, *Allobaculum*, *Enterobacter*, *Enterococcus*, *Proteus*, *Weissella*, and *Bilophila* were increased in the T2DM group, and TBF treatment restored abundances to the levels of the ND group. Additionally, TBFs also increased the relative abundances of *Akkermansia*, *Roseburia*, *Alistipes*, *Odoribacter*, *Blautia*, *Bacteroides*, and *Dehalobacterium*, as shown in Figure 4(c). The level of TNF-*α* was positively correlated with the relative abundance of *Mucispirillum* and the relative abundances of *Akkermansia*, *S24-7*, and *Sutterella* were negatively correlated with HOMA-IR, insulin, and IL-6. The blood glucose level was positively correlated with the relative abundance of *Helicobacter*. The relative abundances of *Helicobacter*, *Clostridium*, and *Lachnospiraceae* were positively correlated with HOMA-IR, insulin, and IL-6 levels and the latter two were negatively associated with HOMA-B and HDL-C, as shown in Figure 4(d). Overall, these findings indicated that the observed TBF effects on microbiota might be associated with metabolic disorders.

## 4. Discussion

Accumulating evidence has suggested that gut microbiota can regulate obesity, diabetes, colon cancer, and nonalcoholic fatty liver disease (NAFLD) [8]. The TBF has shown both antioxidant and antidiabetic effects [26]; hence, dietary intake of TBF can reduce insulin resistance and improve concentrations of blood lipids including TC, TG, LDL-C, and HDL-C in T2DM patients [32]. Rutin and quercetin are the main constituents of Tartary buckwheat and in particular TBF, and both compounds can attenuate diabetes and hence improve blood lipids [17]. However, the effects of TBF on gut microbiota and colon lesions in diabetic mice were not previously investigated. In this work, we determined the effects of TBF on gut microbiota dysbiosis and colon lesions related to diabetes. High dose of STZ causes extensive injury of *β* cells, limiting the secretion of insulin in T1DM mice, and these mice also experience polyphagia and hyperglycemia. The partial injury of *β* cells combined with HFD can impair the function of insulin, resulting in insulin resistance in T2DM mice. In our study, T1DM mice were successfully established, displaying significantly elevated fasting blood glucose, decreased body weight, increased serum LDL-L, total cholesterol (TC), triglyceride (TG), enhanced levels of proinflammatory cytokines, and decreased levels of serum insulin, HDL-C as well. Meanwhile, T2DM mice were successfully established, displaying significantly elevated blood glucose level and decreased body weight, serum LDL-L, total cholesterol (TC), triglyceride (TG), insulin, proinflammatory cytokines, and HDL-C, which are consistent with the results reported in Ref. [25]. The T2DM diabetes is linked to obesity in humans, and T2DM mice were fed HFD and then administered a single injection of STZ to simulate diabetes development [33]. As expected, TBF could effectively improve blood glucose, body weight, lipid profile, insulin, and proinflammatory cytokines in two diabetic models. These results revealed that TBF could be considered a potential adjuvant agent for diabetes. Hyperglycemia and chronic inflammation are closely related to the occurrence of cancer, especially colon cancer and liver cancer [4]. Chronic inflammation can lead to colitis-associated cancer (CAC) and colorectal cancer (CRC), both characterized by the production of proinflammatory cytokines. Certain natural products have anti-inflammatory and anticarcinogenic effects, and some of these products could potentially help protect against inflammation-associated colorectal cancer [34]. However, the potential for TBF to modulate the expression of inflammatory factors that promote intestinal carcinogenesis in diabetic mice has not been evaluated. In our study, TBF significantly decreases the concentrations of serum TNF-*α* and IL-6 in diabetic mice, suggesting that TBF can exert an anti-inflammatory effect to prevent CRC development by reducing inflammatory mediator production. Our results showed that TBF administration rescued damaged villi and crypts, with fewer necrotic sites and vacuolar changes in colon tissues in diabetic mice, suggesting that TBF treatment may reduce the risk of colon cancer. TBF, as secondary metabolites produced by Tartary buckwheat plant, having a variety of potential biological benefits as it was reported that antioxidant, anti-inflammatory, anticancer, antibacterial, and antiviral activity [21]. Because of their unique biological activity and their relative nontoxicity, it is focused by scientists. Further research is needed to improve the availability of flavonoids in diets and thus improve human health. Based on previous experiments [26], we tested low (150 mg TBF/kg body weight) or intermediate (300 mg TBF/kg body weight) doses of TBF in diabetic mice. However, whether these doses are appropriate for human health and if these levels can be attained by dietary intake should be investigated. It is reported that after rutin administration (300 mg/kg) or quercetin administration (30 mg/kg), the Cmax of rutin and quercetin in plasma (0.41 ± 0.08 *μ*g/mL; 1.35 ± 0.37 *μ*g/mL) occurred at 60 min or 30 min (Tmax in plasma), respectively [35]. Gao et al. [36] suggested that colorectal lesions are associated with gut microbiota dysbiosis and this dysbiosis is hence a vital factor in the pathogenesis of CRC [37]. Our results showed that TBF significantly attenuated colon lesions in diabetic mice in a dose-dependent manner, suggesting a potential regulatory effect of TBF on gut microbiota dysbiosis. Overall, the results revealed that TBF exerted antidiabetic effects in the two diabetes models, and these effects may be mediated not through changes in insulin signal-related pathways, but instead may be related to the regulation of gut microbiota dysbiosis. Further research is needed to test the mechanism of action.

Gut microbiota can regulate many metabolic processes, including lipid metabolism, obesity, diabetes, and carcinogenesis. A previous study by Wirth et al. [38] revealed that abnormal gut microbiota in the ileum of T1DM mice might be associated with the development of hyperglycemia, inflammation, pathological microenvironment, and the occurrence of diabetes. Gut microbiota was also found to play an important role in the development of T2DM [39]. Gut microbiota dysbiosis is related to the pathogenesis of obesity and type 2 diabetes [40], with reduced bacterial functional diversity and low community stability in diabetes [41]. We hypothesize that the TBF antidiabetic effects and colon lesions repair in diabetic mice are closely related to the regulation of gut microbiota. As expected, TBF significantly changed the structure of the gut microbiota in diabetic mice, with increased diversity, but the gut microbiota community in the presence of TBF remained significantly different from that of nondiabetic mice. This may be related to insufficient dosage of TBF or an incomplete ability of TBF to alleviate diabetes-related symptoms. Obesity and metabolic syndrome are associated with changes in gut microbiota composition, including increased *Firmicutes*/*Bacteroidetes* (F/B) ratio and increased relative abundance of *Proteobacteria* [40]. A higher abundance of *Firmicutes* and a lower abundance of *Bacteroidetes* in the gut microbiota of diabetic mice are related to hyperglycemia and inflammatory state [42]. In accordance with previous studies, our results demonstrated that TBF effectively decreased *Firmicutes*, increased *Bacteroidetes*, and decreased the ratio of *Firmicutes* to *Bacteroidetes*, suggesting changes in anti-inflammatory species in response to TBF treatment likely explain the observed changes in inflammatory markers. It was reported that increased *Proteobacteria* abundance could promote endotoxin content, affecting the occurrence and development of chronic inflammatory diseases [43]. In this study, TBF reversed the relative abundance of *Proteobacteria* in diabetic mice and increased that of *Actinobacteria*. Thus, TBF effects to lower the *Proteobacteria* abundance and inflammatory state of diabetes mice may contribute to the improvement in glucose homeostasis. Furthermore, previous studies suggested that *Erysipelototrichaeae* and *Streptocaceae* were correlated with the development of obesity, systemic inflammation, and metabolic diseases [44–46]. The high abundance of *Streptocaceae* is related to the risk of inflammation, metabolic disorder, diabetes, and colon cancer [47–49]. In our study, the abundances of *Erysipelototrichaeae* and *Streptocaceae* in T1DM mice were much higher compared to the ND group, and these changes were reversed by TBF. Cani et al. [50] reported a positive relationship between *Escherichia* and the occurrence and development of diabetes and obesity. In this study, TBF reduced the levels of *Helicobacter*, *Lactobacillus*, and *Escherichia* in diabetic mice. Everard et al. [51] reported that an increased F/B ratio might contribute to the pathogenesis of obesity in mice fed a high-fat diet. TBF decreased F/B ratio, decreased the content of *Proteobacteria*, which is closely related to inflammation, and increased the abundance of *Actinobacteria*. The abundances of *Erysipelototrichaeae* and *Streptocaceae* in both T1DM and T2DM mice were higher than those in the ND group, and these differences were significantly reversed by TBF treatment. *Akkermansia* is a mucin-degrading bacterium and can improve insulin resistance [52]. TBF increased *Akkermansia* abundance in T2DM mice, but we observed reduced abundance of *Akkermansia* in the TBF-treated T1DM mice, consistent with improved glucose levels. Although it is easy to speculate that a reduced level of *Akkermansia* contributes to the beneficial effects of TBF in T1DM mice, this contradicts other reports of inverse correlation of *Akkermansia* abundance with glucose levels [53]. Therefore, additional research is required to clarify this relationship. The relative abundances of some bacteria, such as *Bilophila* and *Mucispirillum*, are significantly positively correlated with the development of diabetes [48,54]. The alleviation of diabetes symptoms by TBF may, therefore, be linked to the decreased presence of *Mucispirillum*, *Enterobacter*, *Enterococcus*, *Proteus*, and *Bilophila*. In addition, TBF also increased the presence of bacteria such as *Akkermansia*, *Roseburia*, *Alistipes*, *Odoribacter*, and Bacteroide that impair the development of diabetes. The link between gut microbiota composition and metabolic disease or cancer development remains largely associative and additional research is required to determine whether changes in the composition of gut microbiota are associated with metabolic parameters in STZ-induced T1DM mice and HFD/STZ-induced T2DM mice. Spearman's correlation analysis showed that some key communities including Escherichia, Akkermansia, Helicobacter, Clostridium, and Lachnospiraceae are associated with the main metabolic parameters in diabetic mice. A negative correlation between Sutterella and HOMA-IR, and IL-6 contents was found. Sutterella have anti-inflammation properties [55]. Therefore, the reduction of inflammation by TBF treatment in diabetic mice might be due to the increased abundance of *Sutterella*, and it was also positively correlated with the decrease in serum IL-6, suggesting that TBF effects on microbiota might be associated with metabolic disorders. TBF may affect glycolipid metabolism and colon lesions in diabetic mice by increasing beneficial bacteria and decreasing harmful bacteria. Hartstra et al. [56] emphasized the important role of SCFAs, particularly acetate, propionate, and butyrate, to reduce chronic inflammatory diseases and promote colon cell health. *Roseburia*, *Rikenella*, and *Odoribacter* are butyrate-, propionic acid-, and butyrate-producing intestinal bacteria [57,58]. The SCFAs produced by these microbiotas could provide energy for the host, improve the acidic environment in the colon, inhibit the production of inflammatory factors, and repair mucosal inflammation [49]. In our study, TBF significantly increased the presence of *Alistipes*, *Roseburia*, *Rikenella*, and Ruminococcus, which may increase SCFA production and benefit colon villi in the experimental diabetic mice. The recovery of colon damage by TBF might reflect the enrichment of SCFA-producing bacteria. Targeted recovery of these SCFA producers suggests a new therapeutic approach for patients with diabetes. TBFs exhibited an antidiabetic effect and reduced colon damage in diabetic mice, possibly by regulating the gut microbiota community structure and composition. Thus, the use of TBFs is a potential new strategy to treat diabetic patients, protect intestinal health, and prevent colon cancer carcinogenesis. Further work should investigate the detailed mechanisms of how TBFs and their component flavonoids regulate the gut microbiota in different pathological states.

## 5. Conclusions

In the present study, we demonstrated that the TBF improved blood glucose, body weight, serum lipid profiles (TC, TG, LDL-C, and HDL-C), serum inflammatory factors (TNF-*α* and IL-6) and fasting insulin. The TBF significantly improved diabetes-related colon lesions in diabetic mice. Furthermore, the TBF significantly altered the diversity and composition of gut microbiota. These results indicate that the potential mechanisms of TBF in improving gastrointestinal health and eventually mediating their beneficial effects on diabetes.

## Figures and Tables

**Table 1:** Biochemical parameters of ND mice and T1DM mice with or without TBF treatment after four weeks of treatment.

Group	Blood glucose (mmol/L)	Body weight (g)	TC (mmol/L)	TG (mmol/L)	LDL-C (mmol/L)	HDL-C (mmol/L)	Insulin (mIU/L)
ND	7.13 ± 0.33	21.86 ± 0.70	2.56 ± 0.63	0.75 ± 0.19	0.59 ± 0.17	3.98 ± 1.12	33.52 ± 0.71
T1DM	19.56 ± 3.64^###^	16.50 ± 1.06^#^	4.74 ± 0.99^#^	1.24 ± 0.16^#^	0.92 ± 0.04^##^	1.95 ± 0.56^#^	27.20 ± 1.55^##^
T1DM-TBF150	15.40 ± 0.95^##^	18.40 ± 0.62^*∗*^	2.93 ± 0.93^*∗*^	0.87 ± 0.11^*∗*^	0.67 ± 0.12^∗∗^	4.30 ± 1.30^*∗*^	32.08 ± 2.32^*∗*^
T1DM-TBF300	11.30 ± 1.48^∗#^	19.76 ± 2.1^*∗*^	2.60 ± 0.37^*∗*^	0.76 ± 0.03^∗∗^	0.63 ± 0.11^∗∗^	4.67 ± 0.89^∗∗^	32.98 ± 2.73^*∗*^

^#^ Indicates significantly different from the ND group (one-way ANOVA analysis). Data are expressed as means ± SEM: ^#^*p* < 0.05, ^##^*p* < 0.01, ^###^*p* < 0.001. ^*∗*^Indicates significantly different from the T1DM group (one-way ANOVA analysis). Data are expressed as means ± SEM: ^*∗*^*p* < 0.05, ^∗∗^*p* < 0.01. Normal diet (ND), *n* = 6; Type 1 diabetes mice (T1DM), *n* = 5; type 1 diabetes mice pretreated with 150 mg TBF/kg body weight (T1DM-TBF150), *n* = 5; type 1 diabetes mice pretreated with 300 mg TBF/kg body weight (T1DM-TBF300), *n* = 5.

**Table 2:** Biochemical parameters of ND mice and T2DM mice with or without TBF treatment.

Group	Blood glucose (mmol/L)	Body weight (g)	TC (mmol/L)	TG (mmol/L)	LDL-C (mmol/L)	HDL-C (mmol/L)	Insulin (mIU/L)	HOMA-IR	HOMA-B
ND	8.50 ± 0.87	32.20 ± 1.60	3.14 + 0.51	0.82 ± 0.07	0.84 ± 0.06	4.53 ± 2.00	29.03 ± 2.19	11.32 ± 1.91	112.14 ± 10.80
T2DM	21.0 ± 0.72^###^	26.31 ± 0.85^#^	7.18 ± 0.58^###^	1.63 ± 0.36^#^	4.98 ± 1.55^###^	1.69 ± 0.58^#^	44.10 ± 5.28^#^	40.37 ± 8.28^##^	56.12 ± 4.86^##^
T2DM-TBF150	14.17 ± 1.70^∗∗^^##^	28.80 ± 1.90^*∗*^	3.64 ± 1.59^∗∗^	1.01 ± 0.12^*∗*^	1.89 ± 0.31^∗##^	3.15 ± 0.62^*∗*^	34.32 ± 0.97^∗#^	25.73 ± 3.46^∗#^	75.16 ± 6.64^∗##^
T2DM-TBF300	11.7 ± 1.37^∗∗∗##^	30.90 ± 0.6^∗∗^	2.97 ± 1.03^∗∗^	0.74 ± 0.21^*∗*^	1.77 ± 0.34^*∗*^^##^	6.04 ± 1.78^∗∗^	32.08 ± 2.36^*∗*^	21.30 ± 1.73^∗#^	90.12 ± 11.05^∗∗^

^#^ Indicates significantly different from the ND group (one-way ANOVA analysis). Data are expressed as means ± SEM: ^#^*p* < 0.05,

^##^ *p* < 0.01, ^###^*p* < 0.001. ^*∗*^Indicates significantly different from the T2DM group (one-way ANOVA analysis). Data are expressed as means ± SEM: ^*∗*^*p* < 0.05, ^∗∗^*p* < 0.01. Normal diet (ND), *n* = 5; type 2 diabetes mice (T2DM), *n* = 5; type 2 diabetes mice pretreated with 150 mg TBF/kg body weight (T2DM-TBF150), *n* = 5; type 2 diabetes mice pretreated with 300 mg TBF/kg body weight (T2DM-TBF300), *n* = 5.

**Table 3:** Inflammatory factors of ND mice and T1DM mice with or without TBF treatment after four weeks of treatment.

Group	TNF-*α* (pg/mL)	IL-6 (pg/mL)
ND	17.64 ± 1.64	18.64 ± 5.52
T1DM	47.39 ± 15.05^##^	98.93 ± 29.34^##^
T1DM-TBF150	19.51 ± 1.23^*∗*^	56.99 ± 8.40^∗##^
T1DM-TBF300	17.10 ± 1.17^∗∗^	14.40 ± 10.92^∗∗^

^#^ Indicates significantly different from the ND group (one-way ANOVA analysis). Data are expressed as means ± SEM: ^#^*p* < 0.05, ^##^*p* < 0.01, ^###^*p* < 0.001. ^*∗*^Indicates significantly different from the T1DM group (one-way ANOVA analysis). Data are expressed as means ± SEM: ^*∗*^*p* < 0.05, ^∗∗^*p* < 0.01.

**Table 4:** Inflammatory factors of ND mice and T2DM mice with or without TBF treatment.

Group	TNF-*α* (pg/mL	IL-6 (pg/mL)
ND	18.54 ± 0.57	12.86 ± 5.94
T2DM	23.40 ± 0.86^###^	133.15 ± 46.24^#^
T2DM-TBF150	20.51 ± 1.99^*∗*^	36.05 ± 11.22^*∗*^
T2DM-TBF300	19.71 ± 1.06^∗∗^	19.62 ± 6.13^*∗*^

^#^ Indicates significantly different from the ND group (one-way ANOVA analysis). Data are expressed as means ± SEM: ^#^*p* < 0.05, ^##^*p* < 0.01,

^###^ *p* < 0.001. ^*∗*^ Indicates significantly different from the T2DM group (one-way ANOVA analysis). Data are expressed as means ± SEM: ^*∗*^*p* < 0.05, ^∗∗^*p* < 0.01.

## Data Availability

The data used to support the findings of this study are available from the corresponding author upon request.

## Supplementary Materials

Supplementary Materials Supplementary Figure S1 indicates the HPLC chromatograms of the TBF extract components. Peaks 1 and 2 are identified as rutin and quercetin, respectively. Supplementary Figure S2 indicates sufficient gut microbial diversity was captured in each sample at this sequencing depth in T1DM mice. (a) Rank abundance curve. (b) Species accumulation curves. Supplementary Figure S3 indicates sufficient gut microbial diversity was captured in each sample at this sequencing depth in T2DM mice. (a) Rank abundance curve. (b) Species accumulation curves. Supplementary Figure S4 indicates the Simpson and Shannon index in the *α*-diversity analysis in T1DM and T2DM mice. (a, b) The Simpson and Shannon index in T1DM mice. (c, d) The Simpson and Shannon index in T2DM mice. Supplementary Figure S5 indicates bacterial taxonomic profiling of gut microbiota at the order level in T1DM mice. Supplementary Figure S6 indicates the bacterial taxonomic profiling of gut microbiota at the family level in T1DM mice. Supplementary Figure S7 indicates bacterial taxonomic profiling of gut microbiota at the order level in T2DM mice. Supplementary Figure S8 indicates the bacterial taxonomic profiling of gut microbiota at the family level in T2DM mice.
