# Supplementary material for: Tartary Buckwheat Flavonoids Improve Colon Lesions and Modulate Gut Microbiota Composition in Diabetic Mice
Source: Evid Based Complement Alternat Med. 2022 Aug 16;2022:4524444. doi: 10.1155/2022/4524444 (PMC9398688; doi:10.1155/2022/4524444)
Supplement: Supplementary Materials — Supplementary Figure S1 indicates the HPLC chromatograms of the TBF extract components. Peaks 1 and 2 are identified as rutin and quercetin, respectively. Supplementary Figure S2 indicates sufficient gut microbial diversity was captured in each sample at this sequencing depth in T1DM mice. (a) Rank abundance curve. (b) Species accumulation curves. Supplementary Figure S3 indicates sufficient gut microbial diversity was captured in each sample at this sequencing depth in T2DM mice. (a) Rank abundance curve. (b) Species accumulation curves. Supplementary Figure S4 indicates the Simpson and Shannon index in the α-diversity analysis in T1DM and T2DM mice. (a, b) The Simpson and Shannon index in T1DM mice. (c, d) The Simpson and Shannon index in T2DM mice. Supplementary Figure S5 indicates bacterial taxonomic profiling of gut microbiota at the order level in T1DM mice. Supplementary Figure S6 indicates the bacterial taxonomic profiling of gut microbiota at the family level in T1DM mice. Supplementary Figure S7 indicates bacterial taxonomic profiling of gut microbiota at the order level in T2DM mice. Supplementary Figure S8 indicates the bacterial taxonomic profiling of gut microbiota at the family level in T2DM mice. [file 4524444.f1.zip › Supplementary Figure S2 (1).pdf]

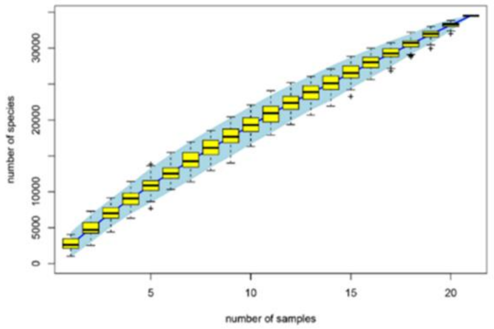

(a)

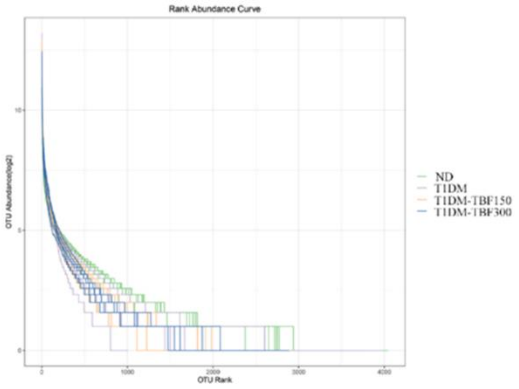

(b)

Supplementary Figure S2 indicates sufficient gut microbial diversity was captured in each sample at this sequencing depth in T1DM mice. (a) Rank abundance curve. (b) Species accumulation curves.
